# Suggestive to Consumptives

**Published:** 1858-11

**Authors:** 


### SUGGESTIVE TO CONSUMPTIVES.

An Editor of one of the most honest and able Christian Reviews in this nation, who consulted us some years ago, for a throat affection, and has been doing good service to the Church for some time past, writes: "I shall ever consider myself greatly indebted to your good advice for much of the comfort and happiness I have enjoyed for some years past. Before I consulted you, I was rapidly becoming a confirmed invalid, afraid to venture out after night, confining myself to the house in all weathers, except the finest and the dryest. Since then, I have braved the winter's storms and summer's rains, and hardly ever think of regarding the weather; and I take cold far less now than when I was so exceedingly careful."

Let our readers remember, we are not housed into health; on the contrary, many, very many persons "muffle themselves up," until, before they are aware of it, they are stepping into the grave. More people die of air-tight rooms, than of unchinked log-cabins.

Some years ago, a young lady from New England consulted us. We did not believe that she had energy enough to undertake a sufficiently active course of life, for the several grave symptoms which presented themselves. She had lately married. Her beauty had been one of the commanding kind; but it had faded away, like a flower without water. Under the circumstances, with a cough that sounded as from a fathomless cavern, we laid down a programme adapted to a long sea-voyage, which peculiar circumstances seemed to make imperatively necessary, whether it was better for her or not. We felt confident she would die in a few months. But there is a persistence in a New Englander's purpose, male or female, when there is a laudable object in view, which defies calculation; and instead of dying, she sends us, at the end of three years, a pithy dictum on the treatment of consumption! It runs thus:

"There is nothing like trying. Sister says I might have been dead, if I had been like some other people. Give my respects to Dr. HALL; and tell him I am very happy that, for once, his anticipations were not realized; though I think, that had I not learned my situation from him, and improved the remedies he advised, I should not have lived. Tell him that, should he ever have a case similar to my own, to tell them to go to sea, to the hottest place in the world, and take care of a husband and a crew of men, all sick with yellow-fever; and when they come home, go to housekeeping, and do their own work. It is the best preventive of consumption I know of; and if it does not cure, it lengthens one's days."

Now, as it so happens, that we have not always a New England girl, all the way from the State of Maine, to begin with, the prescription is an impossibility. Yet, these cases prove several very important facts, to wit: that

*Housing-up will kill any invalid.*

*A person of requisite energy may permanently arrest the progress of consumption anywhere*, North, South, East or West; for IT IS THE OUT-DOOR BODILY ACTIVITIES, AND A WROUGHT-UP MIND, which compels itself away from the contemplation of bodily infirmities, that replaces the hectic with the hue of health, throwing physic to the dogs. How long will it take mankind to learn these patent lessons?

Every year or two brings up a new cure for consumption, and straightway some beautiful theory is fabricated, which fits in so well, that, with one glad accord, it is heralded throughout the land as the conqueror of life's worst enemy; but, by the time people have got into the way of guzzling the filthy stuff down by the gallon, the sage discovery is made, that just as many people die of consumption as before—as, witness the successive rages for Brandy and Salt, Breathing Oxygen, Tar Fumes, Wood Naphtha, Cod-liver Oil, Burnt Bones, and Iron, Medicated Inhalation, Sugar-house Cure; and latest, if not best, is Dr. CHURCHILL'S vaunted remedy, the various Phosphates of Lime, Soda, Iron—everything. Common-sense ought to tell us, that any physician who had a certain remedy for consumption, would, in a year, have the largest fortune on earth; and more glad than all the world besides would the Faculty be, to find such a remedy. As proof, no sooner is a "consumption-cure" proposed, which has any claims to respectability as to source or quality, than it is at once tested and reported upon. Medicated Inhalation having neither, was allowed to die and leave no sign, and is only remembered by those whom it left with life enough to execrate it and its advocates, paid editors, and all. Dr. CHURCHILL'S claims were made with such confidence, the assertions were so unequivocal; names, places, and dates, were so specific; and withal, the remedies themselves were so frankly avowed, that the claims were investigated officially, by the finest and most finished medical minds in Great Britain, with the following very plainly-expressed results:—"As far as we can judge from these cases, it is obvious, that the hypophosphates are of no curative value whatever in the treatment of consumption; indeed, they seem to be simply inert, doing neither good nor harm, except indirectly in the latter sense, by interfering with the more positively beneficial modes of treatment." We, therefore, urge upon our readers the necessity of falling back on first principles—air, food, and exercise; as much of each as can possibly be taken—more fully expressed in a letter written Saturday, March sixteenth, eighteen hundred and forty-four, at New Orleans, by ourselves, to a young physician then living in New York city: "None but the intelligent can have the moral courage requisite to meet, resist, and overcome the constantly recurring back-sets of consumption. After all, I tell you most seriously, that the unstinted use of the fresh, cool air of heaven, is absolutely essential to the lasting benefit or cure of phthisis. Free exposure, without stint; and the more you put yourself to the trouble of thinking about it, the more you will be convinced of its reasonableness. The infinite necessity of an extraordinary amount of air, pure and cool and full of oxygen, is apparent, that by mixing with the carbon of the blood in the lungs, the effete matter, pus, everything, may be segregated and carried out of the body. So I drive my patients out of doors all the time; they must literally live out of doors." Thus we wrote, over fourteen years ago; and with intenser convictions of its truthfulness we re-write it to-day, with those long years of experience to back it. Moderate, continuous bodily activities in the open air, with a mind intensely and pleasurably interested in some highly remunerative pursuit, will cure any case of consumption whose cure is possible; and if this fails, so will all else. When there are complications, with irregular bowels, daily fevers, fullness, or other distress after meals, irregular appetite, shortness of breath, which precludes the necessary amount of exercise with safety—any one of these imperatively requires the constant supervision of a physician of education, experience, and candor. With these conditions, any ordinary case of consumption not in the "advanced stages," may get well anywhere, in Cuba or in Nova Zembla, Summer or Winter, as hundreds of intelligent, energetic men and women have testified, and other hundreds will repeat the testimony now.

At the closing of this article, a young lady writes, on returning from her Summer rambles: "While in the neighborhood, I accompanied a party of friends to the Falls of Niagara, and tired them all out, preferring to walk around Goat Island, instead of riding. On returning at night, I enjoyed a hearty laugh at them, particularly one effeminate city gent. I thought one country lassie worth a dozen such in a walking tour." Two years ago, this lady was laboring under a dry cough, which lasted for hours at a time—debility, night-sweats, and a pulse of a hundred and thirty—and has made herself what she is to-day, almost without medicine, but by the agency of out-door activities, afoot and on horse, carried out with indomitable persistency, even when the thermometer was at zero. Away then, my reader, with physic, and heated rooms, and a southern clime, if the fell destroyer is threatening *you!*

